# How to Evaluate Reports of Intimate Partner Violence? Examining Interpartner Agreement in a Forensic Sample of Different-Sex Couples Where Men are Accused of Intimate Partner Violence

**DOI:** 10.1177/08862605241249740

**Published:** 2024-05-10

**Authors:** Marta Capinha, Daniel Rijo, Marlene Matos, Marco Pereira

**Affiliations:** 1 Center for Research in Neuropsychology and Cognitive and Behavioural Intervention, Faculty of Psychology and Educational Sciences, University of Coimbra, Portugal; 2 Psychology Research Center, School of Psychology, U﻿niversity of Minho, Braga, Portugal

**Keywords:** domestic violence, batterers, battered women, assessment, predicting domestic violence

## Abstract

Research about interpartner agreement on intimate partner violence (IPV) is mainly based on community and clinical samples, with forensic or court-related samples being overlooked. This study assesses interpartner agreement on IPV reports based on the Revised Conflict Tactic Scales, aiming to explore if the proxy method would be reliable in a court-related setting. The study sample comprised 62 different-sex couples identified in the Portuguese judicial system due to an IPV-related crime perpetrated by men. Agreement was assessed based on different indexes: percent agreement and Gwet’s AC1 for occurrence, and Tau-b and intraclass correlations for frequency. Men’s and women’s perpetration were considered. Results showed that interpartner agreement on IPV occurrence (ranging from poor-to-very good) tended to be higher and more consistent among indexes than agreement on IPV frequency (ranging from non-existent to strong). This study highlights the need to collect both partners’ reports in court-related settings.

## Introduction

Intimate partner violence (IPV) is a phenomenon with severe consequences for both victims and families (e.g., Romano et al., 2021; Stöckl et al., 2013), as well as high costs for governments (European Institute for Gender Equality, 2014). A high prevalence of IPV across countries has been identified (e.g., Capinha et al., 2022; Esquivel-Santoveña et al., 2013; European Union Agency for Fundamental Rights, 2014). Nonetheless, the debate around the best way to assess IPV in different settings—namely community, clinical, and forensic settings—is still unsolved. For reasons that range from security and ethical issues to the higher costs associated with gathering both partners reports, the use of a proxy method (i.e., the use of a single partner report) tends to be the most frequent strategy used to assess IPV (Armstrong et al., 2002; Btoush & Campbell, 2009; Moffitt et al., 1997; Straus et al., 1996). If not reliable, trusting in a single partner report (i.e., the use of the proxy method) might lead to wrongly estimated prevalence and frequency rates of violence in the general population, and it may also contribute to erroneous conclusions about interventions’ efficacy, both in clinical as in forensic settings (Heckert & Gondolf, 2000). Consequently, the theoretical rationale for interventions, and even public policies, may also be misguided (Capinha et al., 2024; Moffitt et al., 1997). In forensic settings, assessing the occurrence and frequency of IPV could also impact court decisions, risk assessment, and victims’ protection measures, with enormous consequences for all family members affected by IPV. Therefore, ensuring a structured and reliable method to conduct such assessments is paramount.

In trying to solve the debate around the best method to assess IPV, some authors have focused on evaluating interpartner agreement about IPV. Interpartner agreement is assessed as interrater reliability: if high, it would suggest that both partners agree in their reports of IPV occurrence (or frequency) within their relationship. If that were the case, the proxy method (i.e., using only one of their reports) could, then, be considered reliable (Capinha et al., 2024; Gwet, 2014).

Identified as one of the most widely used instruments to assess IPV, the Revised Conflict Tactic Scales (CTS-2; Straus et al., 1996) has also been the most used instrument to evaluate interpartner agreement within different-sex couples. This instrument has shown good psychometric properties across different samples and countries (e.g., Bell & Naugle, 2007; Paiva & Figueiredo, 2006; Straus, 2004; Straus & Mickey, 2012; Vega & O’Leary, 2007). However, based on the CTS-2, estimates of interpartner agreement within different-sex couples have been mostly identified as ranging from low to moderate, both on the occurrence and frequency of different types of IPV (Caetano et al., 2009; Cunradi et al., 2009; Graña et al., 2017; Marshall et al., 2011). Equivalent findings on interpartner agreement in different-sex couples were observed in specific samples, including war veterans (LaMotte, Taft, Reardon, & Miller, 2014; LaMotte, Taft, Weatherill, et al., 2014), general clinical samples (Simpson & Christensen, 2005), and clinical samples including men with alcohol problems (Panuzio et al., 2006).

The only studies diverging from this low-to-moderate range of findings on interpartner agreement were the research by O’Leary and Williams (2006) and, more recently, by Capinha et al. (2024), both resorting to community samples of different-sex couples. While in the first study, moderate-to-strong agreement levels were found for physical assault, the second study found mostly moderate-to-high agreement levels for all the CTS-2 subscales. Exceptions were the occurrence of psychological aggression (perpetrated by both men and women) and agreement results based on intraclass correlation coefficients (ICC; which identified agreement on frequency as poor for all types of IPV). The authors interpreted the low results on agreement based on this index as a consequence of the parameters influencing its computation. Accordingly, ICC is described as an index of absolute agreement, requiring similarity in the patterns, average, and scatter of both partners’ responses, making it more challenging to achieve a high level of agreement (Furr, 2010). Capinha et al. (2024) also assessed negotiation agreement (the only scale that does not assess violence in the CTS-2). Findings pointed to agreement levels ranging mainly from moderate to strong on its frequency, but agreement slightly decreased when only community couples who reported at least one IPV behavior during the past year were analyzed. The agreement on negotiation occurrence was very good, without differences identified between men and women.

Indeed, existing findings are limited and inconclusive regarding the association between interpartner agreement and gender (men vs. women) or role (victim vs. perpetrator) in the IPV dynamic. While some authors found that both men and women tend to underreport their perpetration of IPV, compared to the report of victimization by their partners (O’Leary & Williams, 2006; Simpson & Christensen, 2005), others found a slightly higher level of agreement regarding men’s perpetration of physical assault (although still poor; Cunradi et al., 2009). Nonetheless, other studies failed to find any association between gender or role (victim vs. perpetrator) and interpartner agreement (Marshall et al., 2011; Moffitt et al., 1997).

### Interpartner Agreement in Forensic and Court-Related Samples

Forensic samples, particularly those from criminal courts, represent one of the most relevant groups to study for those aiming to achieve a deeper understanding of IPV, or at least, its more recurrent and severe forms, including the most severe injuries resulting from physical assault (Cares et al., 2024; Johnson, 1995, 2008). Yet, forensic samples have been an exception in research focusing on interpartner agreement. To the best of our knowledge, only two studies using samples collected in legal settings examined interpartner agreement on IPV. In the first study, Heckert and Gondolf (2000) assessed agreement on the occurrence of physical assault perpetrated by men within couples in which they were court-ordered to IPV treatment programs. Men’s current partners were evaluated, regardless of being the victims or a new partner. In this study, the original version of CTS (Straus, 1979) was used to estimate the percentage agreement at treatment intake and follow-up, but only violence perpetrated by men was assessed. Three forms of percent agreement were calculated: (a) total agreement (dividing the number of cases in which both partners reported the occurrence of physical assault by the total of cases); (b) occurrence agreement (dividing the number of cases in which both partners reported the occurrence of physical assault by the number of cases in which at least one partner had reported physical assault); and (c) male denial (percentage of men who did not report the occurrence of physical assault when women did). At intake, authors found a total agreement of 63.8%, occurrence agreement was 61.4% and male denial was 18.9%. At follow-up, these percentages were 73.5%, 17.3%, and 79.6%, respectively. This means that follow-up contains both the highest percentage of total agreement and the lowest percentage of occurrence agreement, and that male denial dramatically increased from intake to follow-up. These findings should be interpreted in the light of the knowledge that percent agreement indexes do not account for chance (Simpson & Christensen, 2005) and could be biased by different rates of occurrence, as the authors recognized (Heckert & Gondolf, 2000). Moreover, the samples collected at intake (*n* = 149) and follow-up (*n* = 558) were not the same. Heckert and Gondolf (2000) also performed qualitative evaluations of verbal descriptions of the assaults by men and women, as well as the police reports. Findings indicated that both partners could underreport IPV. The authors then concluded that, when assessing interventions’ efficacy, or the prevalence of IPV, reports of both partners should be included, as the report by uniquely one of the partners could not be considered entirely reliable. Using a different instrument (the Abusive Behavior Inventory), a recent study by Stover et al. (2022) assessed both men’s and women’s perpetration in a sample of 282 co-parents referred to an IPV intervention and came to similar conclusions. Based on the ICC, poor levels of agreement were found (ranging from .12 for men’s perpetration of physical abuse to .32 for women’s perpetration of psychological abuse). ICC was also slightly higher for overall women’s perpetration (ICC = .34) than for men’s perpetration (ICC = .18). Women reported higher IPV use by themselves and by their co-parents.

It is worth noting that both studies collected samples of participants under a court-ordered form of treatment. As treatment programs tend to have specific inclusion criteria, the variability of these couple/dyads’ characteristics could be constrained and could impair the extrapolation of data to other legal settings. In addition, both studies relied on a single form of agreement index, a practice not aligned with the recommendations for the study of interpartner agreement (Armstrong et al., 2002; O’Leary & Williams, 2006; Yoshikawa et al., 2021). Finally, these studies focused only on physical assault (both studies) and psychological abuse (Stover et al., 2022). No data were collected to assess interpartner agreement regarding other forms of IPV, although it is known that physical assault often occurs concurrently with other forms of IPV (Krebs et al., 2011; Marshall & Holtzworth-Munroe, 2002). These studies are, nonetheless, important exceptions in the interpartner agreement literature, as data on forensic samples is almost nonexistent.

To the best of our knowledge, no studies used the CTS-2 to analyze interpartner agreement within forensic or court-related samples. This would be particularly important because: (a) the CTS-2 is probably the most widely used self-report instrument to assess IPV in community settings, at least in the West (Straus & Mickey, 2012) and assert its reliability in forensic settings could allow for broader use of this instrument, as well as to enhance the exchange of knowledge between settings while contributing to a deeper understanding of the full range of the IPV phenomenon; (b) the CTS-2 has shown non-existent or low negative correlations with social desirability (Bell & Naugle, 2007; Straus & Mickey, 2012), a particularly relevant concern in legal forensic settings; (c) some research indicated that different levels of violence severity (expected to occur when comparing community and forensic samples) may yield different levels of agreement (Cunradi et al., 2009); (d) the CTS-2 allows for the assessment of patterns of IPV perpetration and victimization within relationships and potential associated risks (e.g., the presence and severity of behaviors or the presence of a bidirectional pattern); (e) in practical terms, it can assist in collecting evidence and informing relevant judicial and probation decisions (e.g., coercive measures, protective measures, co-parenting management) through the analysis of a consistent history of behaviors; and (f) it can inform probation and clinical settings by aiding in the planning of appropriate clinical/forensic psychological interventions (e.g., rehabilitation, prevention).

Acknowledging the criticism regarding the CTS-2 for not accommodating all forms of IPV (e.g., excluding stalking and controlling behaviors) nor the motives behind the use of violence (Kimmel, 2002), the expertise of clinical/forensic psychologists would be fundamental in the interpretative analysis of this assessment. They understand deeply the complexity of evaluating abusive intimate contexts and based their evaluation on a multi-method approach, triangulating information sources. Additionally, there is the assumption that legal settings could be more prone to the existence of different motivations to intentionally distort or conceal the information in the reports (Moffitt et al., 1997), especially if these reports could influence decisions about restraining orders or even child custody (O’Leary & Williams, 2006). These reasons add to other factors already identified as possible influences on IPV reports and interpartner agreement (e.g., memory, education, social desirability, relationship satisfaction; Armstrong et al., 2002; Marshall et al., 2011; Yoshikawa et al., 2021). Furthermore, challenges that arise when trying to assess both partners (Straus et al., 1996), including concerns with safety and ethical issues (Btoush & Campbell, 2009; Ellsberg & Heise, 2002), are even stronger in legal settings and usually surpass the benefits of collecting self-report data from both partners. This could justify the scarcity of research in these settings, but it does not diminish its necessity or relevance.

### The Present Study

This study assessed interpartner agreement in a forensic sample based on the CTS-2. To do so, the methodology followed by Capinha et al. (2024) (which assessed agreement in a Portuguese community sample) was used to investigate if findings from a community sample of couples would be replicated in a different (court-related) sample. However, with so little research available resorting to forensic samples, hypotheses regarding the expected results could take several forms. On one hand, literature seems to point to a higher distrust in the reports of couples from forensic samples (e.g., Moffitt et al., 1997; O’Leary & Williams, 2006). Thus, the agreement could be expected to be lower than what was previously found in the community (Caetano et al., 2009; Capinha et al., 2024; Cunradi et al., 2009; Graña et al., 2017; Marshall et al., 2011) and clinical samples (Panuzio et al., 2006; Simpson & Christensen, 2005). On the other hand, findings from previous studies that used different instruments with forensic samples pointed to low (Stover et al., 2022) or low-to-moderate (Heckert & Gondolf, 2000) levels of agreement. These findings are similar to what has been found in community samples. Accordingly, an equally valid hypothesis would be to expect agreement in this study sample to be similar to those previously found in community samples. Finally, considering that more prevalent and more severe IPV has been associated with higher agreement (Cunradi et al., 2009; O’Leary & Williams, 2006) and that forensic samples are expected to show more prevalent and more severe IPV behaviors (Cares et al., 2024; Johnson, 1995, 2008), it is also possible to anticipate a higher agreement level in this study, compared with findings from community samples.

## Method

### Participants and Procedures

This study was conducted following the Declaration of Helsinki and considered the specific recommendations for research in IPV (Btoush & Campbell, 2009; Ellsberg & Heise, 2002). Ethical approval was obtained from the Ethics Committee of the host institution, and the National Directorate of Prison Services and Social Reintegration (Prison and Probation Services) of the Ministry of Justice of the Portuguese Government. Inclusion criteria were: (a) being in a different-sex intimate relationship, married or cohabitating for at least 3 months; (b) both partners agreeing to participate; (c) both being older than 18 years old; (d) at least one of them should be Portuguese; if one of them was not, he/she must speak Portuguese fluently; (e) no partner reporting to have been diagnosed with a psychotic disorder; (f) the man having a probation measure due to a crime of domestic violence toward his current partner, which may entail a suspended prison sentence or provisional suspension of criminal procedures; and (g) neither the perpetrator nor the victim had yet participated in any therapeutic IPV intervention. Only different-sex couples in which the man was identified in the court case as the perpetrator were considered eligible because of the prior knowledge that most reports of this crime fall on men involved in this type of intimate relationship (in Portugal, over 80% of defendants in domestic violence crimes are usually men; Sistema de Segurança Interna, 2021). For these reasons, the terms men and perpetrators, as well as women and victims, will be used interchangeably throughout this article as they refer to the participant’s role in the judicial process.

The sample was recruited by convenience, both in city and countryside areas. Couples were first contacted by the probation officer responsible for the supervision and monitoring of all obligations (injunctions or rules of conduct) associated with the probation measure. Whenever possible, victims and perpetrators were contacted separately, to avoid any coercion about the participation. They were invited to participate in a study about people whose problems in intimate relationships led to a probation measure. Concurrently, they were assured about the voluntary nature of participation, the confidentiality and anonymity of the data, and the absence of any impact on the probation measure, regardless of whether any of them agreed to participate or not. If both the victim and perpetrator agreed to participate, a date was set to conduct the assessment with the researcher. Perpetrators and victims were assessed separately, but one immediately after the other, not allowing for any discussion about what was asked. When they met the researcher, after being informed about the goals of the study and the confidentiality and anonymity of the data being again guaranteed, all participants provided written informed consent for their participation. All participants completed the CTS-2 and other measures comprising the research protocol (not relevant to this study). The researcher who conducted the assessments had more than a decade of experience in working with perpetrators and victims of domestic violence. By the end of the assessment procedure, victims were informed about victim support organizations available in their residence area.

The sample was comprised of 62 couples (*n* = 124), aged between 21 and 81 years old. Most couples were married (59.7%), had one or two children (68.5%), and lived in rural areas (54.8%). Only 4.8% of participants had a nationality other than Portuguese (mainly from CPLP—Community of Portuguese Speaking Countries). Men were in their 50s (*M* = 51.23; *SD* = 12.81) and women were in their late-40s (*M* = 48.92; *SD* = 12.18). The majority of men (71.0%) had between 5 and 10 years of education, whereas the majority of women (64.5%) had nine or fewer years of education. Most men and women did not consider themselves financially dependent on their partners (72.6% and 67.7%, respectively). Differences were significant regarding age (*z* = −3.17, *p* = .002) but not financial dependence (χ^2^(1) = .29, *p* = .590).

### Measures

The Revised Conflict Tactic Scales (CTS-2; Straus et al., 1996; Portuguese version by Paiva & Figueiredo, 2006) is a 78-item self-report questionnaire that measures physical assault, psychological aggression, injury, and sexual coercion inquired from the perspective of the perpetrator (“I did it to my partner”) and from the perspective of the victim (“My partner did it to me”). These items also assess the use of negotiation strategies by the respondent and his/her partner. Items scale range from (1) “Once in the last year” to (6) “More than 20 times in the last year,” including the options (7) “Not in the last year but have occurred previously,” and (8) “Never occurred.” Occurrence is calculated based on the presence of the behavior during the past year (items scored from 1 to 6), and throughout the relationship (items scored from 1 to 7). Frequency is computed using the mean point of each score, according to the guidelines of Straus et al. (1996). For this sample, reliability, based on Cronbach’s alpha, was .86 and .81 for physical assault victimization and perpetration, respectively; .62 and .79 for psychological aggression perpetration and victimization; .51 and .41 for injury perpetration and victimization; .77 and .67 for sexual coercion perpetration and victimization; and .75 and .73 for negotiation used by the participant and used by the partner, respectively. Despite injury victimization and perpetration missed the cut-off usually defined to be considered minimally acceptable (>.60; Hair et al., 2015; Nunnally & Bernstein, 1994), they were still included in the analysis as the purpose of the present study was to analyze a different form of reliability of these subscales (i.e., the interrater reliability) and no data about it are currently known. Nonetheless, it must be noticed that agreement upon injury could be impaired by these low reliabilities (O’Leary & Williams, 2006).

### Analytical Procedure

As stated above, analytical procedures were similar to those implemented by Capinha et al. (2024). Therefore, the recommendations about the use of different agreement indexes to assess interpartner agreement (Armstrong et al., 2002; O’Leary & Williams, 2006; Yoshikawa et al., 2021) and the preferable use of Gwet’s AC1 instead of Cohen’s Kappa (Dettori & Norvell, 2020; Gwet, 2014; Konstantinidis et al., 2022) were followed. Similarly, the occurrence (i.e., prevalence) and frequency of IPV behaviors were also explored to contextualize the results from interpartner agreement. SPSS STATISTIC (Version 22; IBM Corp., Armonk, NY) was used to compute descriptive statistics, Cronbach’s alpha, means, and proportions comparisons, and Kendall’s Tau-b correlations (as an index of agreement on IPV frequency, i.e., based on continuous variables). Gwet’s AC1, percent agreement, and percent agreement by chance indexes (as indexes of agreement on IPV occurrence, i.e., based on categorical variables, using irrCAC Package), as well as ICC estimates and their 95% confidence intervals (as another index of IPV frequency agreement, using the one-way random effects model from irr Package), were computed using Rstudio (Version 1.4.1717; RStudio, PBC, Boston, MA).

Gwet’s AC1 ranges between 0 and 1, with 1 representing perfect agreement. It was interpreted using a standardized method of benchmarking proposed by Gwet (2014), which consists of the computation of the cumulative probability (CumProb) of an agreement coefficient falling within a given benchmark range. The Altman’s benchmark scale presented by Gwet (2014) was used (i.e., <.20 = poor; .21–.40 = fair; .41–.60 = moderate; .61–.80 = good; .81–1.00 = very good). The recommended threshold of .95 was applied, meaning that the AC1 estimates were interpreted as being in the first range with a CumProb equal to or higher than .95. Direct comparisons with a given benchmark scale could misguide conclusions. It would also not allow for comparisons between studies, as they could depend on sample size and answers distribution (Dettori & Norvell, 2020; Gwet, 2014; Konstantinidis et al., 2022; for an overview of the advantages of Gwet’s AC1 and this method of benchmarking in the context of interpartner agreement on IPV, see Capinha et al., 2024). Tau-b ranges between −1 and 1, with 1 representing perfect agreement. Its interpretation followed the guidelines suggested by Botsch (2011; i.e., <.10 = very weak; .10–.19 = weak; .20–.29 = moderate; ≥.30 = strong, whether they represented agreement or disagreement). ICC usually ranges between 0 and 1, with 1 representing perfect agreement. Nonetheless, negative values may occur, being interpreted as poor agreement (Giraudeau, 1996). Its interpretation followed the Koo and Li (2016) guidelines (i.e., <.50 = poor; .5–.75 = moderate; .75–.90 = good; >.90 = excellent reliability).

## Results

### Occurrence of Different Types of IPV and Negotiation Strategies, Reported by Men and Women: Throughout the Relationship and During the Past Year

The occurrence of different types of IPV and negotiation strategies throughout the relationship and during the past year, distinguishing perpetration by men (perpetrators) and by women (victims), is presented in Table 1. Regarding past-year perpetration by men, men tended to report slightly less occurrence of IPV than women, except for psychological aggression (reported as occurring slightly more by men). Women tended to report lower occurrence of women’s perpetration of physical assault and psychological aggression, but higher women’s perpetration of sexual coercion.

**Table 1.** Occurrence of Different Types of IPV and Negotiation Strategies Throughout the Relationship and During the Past Year.

Type of Violence	Men Reports		Women Reports	
Relationship	Men P (%)	Women P (%)	Men P (%)	Women P (%)
Physical assault	95.2	71.0	93.5	61.3
Psychological aggression	100	95.2	100	98.4
Injury	74.2	27.4	79.0	27.4
Sexual coercion	30.6	11.3	35.5	14.5
Adaptive strategies	MU (%)	WU (%)	MU (%)	WU (%)
Negotiation	98.4	100	90.3	98.4
Past Year	Men P (%)	Women P (%)	Men P (%)	Women P (%)
Physical assault	32.3	30.6	35.5	25.8
Psychological aggression	77.4	74.2	74.2	69.4
Injury	12.9	6.5	21.0	6.5
Sexual coercion	19.4	4.8	25.8	8.1
Adaptive strategies	MU (%)	WU (%)	MU (%)	WU (%)
Negotiation	93.5	95.2	87.1	95.2

*Note*. Men P = men’s perpetration; Women P = women’s perpetration; MU = men’s use; WU = women’s use.

### Frequency of Different Types of IPV and Negotiation Strategies During the Past Year, Reported by Men and Women

Frequencies of men and women’s perpetration of different types of IPV and the use of negotiation strategies are presented in Table 2. Men and women tended to report their perpetration of IPV at lower rates than their partners reported being victimized, except for women’s perpetration of sexual coercion. Regarding the use of negotiation strategies, both perpetrators and victims reported using them more frequently than their partners reported they do.

**Table 2.** Frequency of Different Types of IPV and Negotiation During the Past Year.

Type of Violence	Men Reports		Women Reports	
	Men P	Women P	Men P	Women P
	*M* (*SD*)	*M* (*SD*)	*M* (*SD*)	*M* (*SD*)
Physical assault	4.05 (14.26)	3.66 (14.95)	10.48 (27.04)	1.05 (2.29)
Psychological aggression	20.23 (24.34)	18.40 (23.66)	35.69 (39.52)	17.68 (20.29)
Injury	0.66 (2.86)	0.23 (1.41)	0.90 (2.93)	0.15 (0.65)
Sexual coercion	1.15 (3.64)	0.16 (0.75)	5.66 (15.27)	2.00 (11.22)
Adaptive strategies	MU	WU	MU	WU
Negotiation	42.37 (32.65)	40.29 (31.33)	32.68 (33.28)	51.94 (37.64)

*Note*. Men P = men’s perpetration; Women P = women’s perpetration; MU = Men’s use; WF = women’s use.

### Interpartner Agreement on the Occurrence of Different Types of IPV and Negotiation: Throughout the Relationship and During the Past Year

Interpartner agreement concerning the occurrence of IPV throughout the relationship and during the past year was mostly moderate concerning percent agreement and AC1 (see Table 3). Relying on percent agreement and concerning perpetration by men, agreement on physical assault and psychological aggression was higher for a relationship than past-year occurrence, and the opposite was observed regarding the agreement on sexual coercion and injury. Regarding perpetration by women, percent agreement on physical assault and injury was lower for relationship occurrence, whereas agreement on psychological aggression was higher for past-year occurrence. The highest agreement levels (regarding both percent agreement and AC1) were found for negotiation (past-year and throughout the relationship), used by either partner. The exception was the agreement on the use of negotiation by men throughout the relationship, which was lower than the agreement on men’s perpetration of physical assault and psychological aggression.

**Table 3.** Interpartner Agreement on the Occurrence of Different Types of IPV and Negotiation Throughout the Relationship and During the Past Year.

Type of violence	Percent Agreement		Percent Chance Agreement		AC1 (Standard Error)			
Relationship occurrence	Men P	Women P	Men P	Women P	Men P		Women P	
Physical assault	0.95	0.71	0.11	0.45	.95*** (.03)	Very good (1.00)	.47*** (.12)	Fair (.99)
Psychological aggression	1	0.97	1	0.06	1.00	—	.97*** (.02)	Very good (1.00)
Injury	0.82	0.77	0.36	0.40	.72*** (.09)	Moderate (1.00)	.62*** (.10)	Moderate (.99)
Sexual coercion	0.66	0.81	0.44	0.22	.39** (.12)	Poor (1.00)	.75*** (.08)	Good (.97)
Adaptive strategies	MU	WU	MU	WU	MU		WU	
Negotiation	0.92	0.98	0.11	0.02	.91*** (.04)	Very good (1.00)	.98*** (.02)	Very good (1.00)
Past year occurrence	Men P	Women P	Men P	Women P	Men P	Range (CumProb)	Women P	Range (CumProb)
Physical assault	0.71	0.79	0.45	0.41	.47*** (.12)	Fair (.99)	.65*** (.10)	Moderate (.99)
Psychological aggression	0.77	0.79	0.37	0.41	.64*** (.10)	Moderate (.99)	.65*** (.10)	Moderate (.99)
Injury	0.82	0.90	0.28	0.12	.75*** (.08)	Good (.97)	.89*** (.05)	Very good (.97)
Sexual coercion	0.77	0.87	0.35	0.12	.65*** (.10)	Moderate (1.00)	.85*** (.06)	Good (1.00)
Adaptive strategies	MU	WU	MU	WU	MU		WU	
Negotiation	0.90	0.94	0.17	0.09	.88*** (.05)	Very good (.95)	.93*** (.04)	Very good (1.00)

*Note*. Agreement for lifetime prevalence use of male-to-female psychological abuse was perfect (100%), so *p*-value, standard error, confidence interval, and cumulative probability of agreement were not computed. Men P = men’s perpetration; Women P = women’s perpetration; CumProb = cumulative probability of an agreement; MU = men’s use; WU = women’s use.

** *p* < .01. ***p < .001.

### Interpartner Agreement on the Frequency of Different Types of IPV and Negotiation During the Past Year

Interpartner agreement about the frequency of different types of IPV and negotiation strategies during the past year are presented in Table 4. Based on ICC, agreement was poor (<.50) for all IPV types and negotiation subscales, except for injury perpetrated by women (ICC = .58, which can be considered moderate). Based on Tau-b, the agreement was strong (≥.30) for negotiation and all types of violence perpetrated by men. For IPV perpetrated by women, Tau-b was only strong concerning physical assault (Tau-b = .51, *p* < .001) and psychological aggression (Tau-b = .48, *p* < .001). The agreement was not significant for women’s perpetration of injury and sexual coercion nor women’s use of negotiation.

**Table 4.** Interpartner Agreement on the Frequency of Different Types of IPV During the Past Year.

	ICC				Tau-b Correlation	
Type of violence	Men P	CI (95%)	Women P	CI (95%)	Men P	Women P
Physical assault	.23	[−.02, .45]	.08	[−.17, .32]	.41***	.51***
Psychological aggression	.40	[.17, .59]	.47	[.26, .65]	.46***	.48***
Injury	.16	[−.09, .39]	.58	[.38, .72]	.38**	.22
Sexual coercion	.08	[−.17, .32]	−.01	[−.26, .24]	.37**	−.07
Adaptive strategies	MU	CI (95%)	WU	CI (95%)	MU	WU
Negotiation	.39	[.16, .58]	.21	[−.04, .43]	.31***	.15

*Note*. ICC = intraclass correlation coefficients; Men P = men’s perpetration; Women P = women’s perpetration; MU = men’s use; WU = women’s use; CI = confidence interval.

** *p* < .01. ***p < .001.

## Discussion

Research offering empirical-based knowledge about the assessment of IPV in legal settings is of paramount importance. The present study investigates interpartner agreement between perpetrators (men) and victims (women) of IPV in a forensic sample. All subscales of the CTS-2 (different types of violence and negotiation) were analyzed, considering the use of violence by perpetrators and victims. Findings demonstrate different levels of agreement on the perpetration of IPV by men and women, and concerning the different types of violence. Whether perpetrated by men or women, in the past year, the occurrence of injury gathers the highest agreement, whereas psychological aggression and physical assault have the highest agreement on their frequency. When analyzing IPV episodes throughout the relationship, the highest agreement is achieved for psychological aggression.

The present study is a major and relevant contribution to the existing literature, being the first to report interpartner agreement using the CTS-2 in a forensic sample applying multiple index agreements in doing so, including the AC1 (known to outperform the usually chosen Cohen’s Kappa; Dettori & Norvell, 2020; Gwet, 2014; Konstantinidis et al., 2022). Even though the CTS-2 has been extensively used in large community sample research (e.g., Caetano et al., 2009; Capinha et al., 2022; Straus & Mickey, 2012), evidence regarding its reliability in forensic settings may contribute to standardizing procedures. This standardization would facilitate the comparison of data across different settings and countries. A common instrument (i.e., also used in community settings) would also pose an advantage to researchers, allowing for a deeper understanding of IPV. In addition, being able to consider the CTS-2 a reliable instrument, even in the presence of only one of the partners’ reports, may assist probation officers in tailoring their risk assessment and injunctions to the specific characteristics of each case. This study also represents an advance on current knowledge by being the first study to include the assessment of agreement on negotiation and agreement on IPV occurrence throughout the relationship in a sample from a legal setting.

In this study, agreement on past-year occurrence of IPV tends to be slightly higher compared with previous findings on interpartner agreement on IPV within community and clinical samples: it ranges from moderate to good for men’s perpetration (except for physical assault, which yields only fair agreement), and from a moderate to very good for women’s perpetration. Findings from the indexes regarding agreement on the frequency of IPV are inconsistent, as ICC suggests low agreement and Tau-b suggests strong agreement. Nonetheless, lower agreements could be expected from the ICC as this is an index of absolute agreement (Capinha et al., 2024; Furr, 2010), and the use of different couples as raters lead to a higher rating variability (i.e., a higher rater effect; Gwet, 2014).

The same pattern of higher agreement on the occurrence rather than on the frequency of the reported behaviors was also found in this study concerning the use of negotiation strategies. These findings suggest that victims and perpetrators are more likely to agree about the occurrence (throughout the relationship or during the past year) of positive behaviors, rather than the occurrence of IPV, but not necessarily about the frequency of IPV or the resort to negotiation strategies. As perpetrators and victims report using negotiation strategies more frequently than their partners recognize they use them, it could be important to assess if this represents an attempt (intentional or not) to justify or rationalize their use of violence. Future research should address this topic to investigate if this pattern of findings would be replicated.

Comparing the findings of the present study with those from the community sample analyzed by Capinha et al. (2024), they cannot be considered replicated in the present work. Regarding the past-year occurrence of IPV, the tendency for a lower interpartner agreement in the forensic sample suggests that resorting to uniquely one partner report would be a more reliable strategy in non-legal settings. However, if comparisons are made with other studies focusing on community samples (e.g., Heckert & Gondolf, 2000; Stover et al., 2022) this conclusion may not be drawn, as those studies’ findings also pointed mainly to a lower agreement level than those found by Capinha et al. (2024). Therefore, the use of different indexes (namely Gwet’s AC1 instead of Cohen’s Kappa, and ICC in addition to Tau-b) could explain these discrepancies, calling for the need for replication and to incorporate of more reliable indexes in future research.

Regarding the agreement on the frequency of IPV, overall, comparable agreement ranges were found in the present study and the former study by Capinha et al. (2024). Nonetheless, couples in the present study (i.e., from a legal setting) seem to agree more on men’s perpetration of psychological aggression frequency and women’s perpetration of psychological aggression and injury. Relying on Tau-b, agreement on frequency was higher in this study sample for all types of perpetration by men, and for perpetration of physical assault and psychological aggression by women. This could be explained, at least partially, by the higher prevalence of these behaviors within the forensic sample compared to the community sample (O’Leary & Williams, 2006). Moreover, the existence of more severe behaviors, therefore easier to remember, may make it easier to report them. This could contribute to the similarity in the pattern of reports from both partners (assessed by Tau-b), but also increase similarity on their means and scatter of reports (comprised in ICC; Furr, 2010), leading to higher agreement on frequency in the forensic sample.

The novelty of assessing negotiation agreement, which is done in both Capinha et al. (2024) and the present study, also allows for comparisons, and it shows the same range (very good) of agreement in both studies. This may indicate that partners in both legal and community settings are equally likely to agree when reporting the occurrence of positive behaviors (throughout the relationship and during the past year), but not the occurrence nor frequency of negative (i.e., violent) ones.

The findings from this study also suggest a greater challenge in untangling the understanding of agreement in legal settings, than just assuming that couples would deliberately under or overreport IPV. In community and clinical samples, some variables are identified as being potentially able to influence both partners’ reports (e.g., the ability to recall a given event or placing that event in a specific period, education level, or even the interpretation of the questions, self-justification, relationship satisfaction), and there is a belief that men and women, or victims and perpetrators, might have different report styles (Armstrong et al., 2002; Graña et al., 2017; Marshall et al., 2011; Moffitt et al., 1997; Simpson & Christensen, 2005; Yoshikawa et al., 2021). For example, one may hypothesize that those who perpetrate IPV may be more prone to be influenced by self-justification or that women presenting higher relationship satisfaction are less prone to report/interpret some behaviors as violence (Graña et al., 2017). Nonetheless, findings have been inconsistent and variations in reports do not seem to be associated with gender or the role of victim versus perpetrator (Capinha et al., 2024; Marshall et al., 2011; Moffitt et al., 1997). Therefore, this should be an avenue for further research, considering both individual and relational variables.

When comparing current findings with those of other studies analyzing samples from legal settings, the percent agreement on men’s perpetration of physical assault (71%) from this study was higher than the total agreement identified by Heckert and Gondolf (2000) at the beginning of intervention (63.8%), but lower than the agreement identified at follow-up (73.5%). However, all these values indicate that under three-quarters of participants give similar reports, not accounting for agreement by chance. Compared to the agreement levels found by Stover et al. (2022), the present study results showed higher agreements on the perpetration of psychological aggression by men (0.40 vs. 0.21) and by women (0.47 vs. 0.32, respectively), and on the perpetration of physical assault by men (0.23 vs. 0.12). Regarding perpetration by women, agreement on psychological aggression was higher on the study by Stover et al. (2022) than in the current one (0.08 vs. 0.29). These authors also assessed the agreement on specific items focusing child and home-related power and control tactics, deemed as an advantage over CTS-2, but the levels of agreement were interpreted as “as good as random” (p. 11). This implies the ineffectiveness of these items if only one partner report is available, although the authors advocate for the use of this instrument based on this characteristic.

In the present study, the most prevalent behaviors reported (psychological aggression during past-year, and psychological aggression and physical assault throughout the relationship) are also those that generally gather higher agreement on their occurrence, in line with the idea that more prevalent behaviors could lead to the higher agreement (O’Leary & Williams, 2006). These types of violence are also the ones reported as more frequent during the past year (except for the reports by women of their perpetration of sexual coercion), albeit they are not those showing the most agreement regarding frequency. This could be because it may be more difficult for couples to remember how often a given behavior occurred than to remember if it did occur (Simpson & Christensen, 2005). Additionally, as previously mentioned, agreement levels tend to be better and more consistent regarding IPV occurrence than IPV frequency. Therefore, couples in the forensic sample analyzed in the present study seem to have been more reliable in reporting what type of IPV behavior had occurred between them, rather than how often those behaviors occurred. This evidence finding agrees with the idea that differences in the perception and recollection of past events of IPV are a more probable explanation concerning the (not so high) levels of agreement, than with the idea that those differences come from a conscious attempt to manipulate or conceal information, usually associated with legal settings. Previous research showing that CTS-2 is not associated with social desirability (Bell & Naugle, 2007; Straus & Mickey, 2012) reinforces this hypothesis. This hypothesis is also in line with findings in this study showing that both victims and perpetrators were consistent in their tendency to report that more violence (occurrence and frequency) is used by men (the partner identified as the perpetrator by the legal system). Finally, it should also be noted that the assessment of frequency could be influenced by the type and duration of probation measures as will be discussed below.

Regarding past-year occurrence, this study’s findings show that partners tend to reveal higher agreement on perpetration by women than perpetration by men, with the opposite tendency (higher agreement on perpetration by men than on perpetration by women) emerging for the occurrence throughout the relationship, except for sexual coercion. A similar trend for higher agreement regarding women’s perpetration was found for the agreement about the frequency of IPV during the past year. This means that victims’ (women) use of violence (either occurrence or frequency) during the past year, tended to garner more accord than the use of violence by perpetrators (men). This could be because men were under probation measures at the time of data collection and, therefore, were more prone to underreport their aggressive behavior. Although couples were beyond a phase of prosecution (and/or seeking help), and full confidentiality and the strict research goals of their participation had been guaranteed, they might still have been hesitant to fully share their IPV experiences. Hence, perpetrators could be underreporting their use of violence during the past year (in both occurrence and frequency), perhaps fearing legal consequences from the disclosure, as has been suggested (Heckert & Gondolf, 2000; Moffitt et al., 1997). It can also be the case that victims overreported their partners’ use of violence while underreporting their own, particularly regarding frequency, to reinforce the pertinence and legitimacy of the probation regimen or to try getting (or not lose) help (Moffitt et al., 1997). Unlike other studies in legal settings (Heckert & Gondolf, 2000; Stover et al., 2022), this study was not associated with any intervention. Therefore, the motivation to participate in an intervention or to undermine that participation would not have played such an influential role.

In the present study, only women’s perpetration of sexual coercion is more reported by women themselves than by men (in occurrence and frequency). Sexual coercion emerging as an exception compared to other types of IPV could be due to the ambiguous nature of some items (Caetano et al., 2009; Simpson & Christensen, 2005), which may lead women to identify neutral behaviors as coercive or men to identify coercive behaviors as neutral. Indeed, traditional gender roles might help to explain a benevolent interpretation of certain coercive sexual behaviors perpetrated by men but, on the other hand, they might also contribute to a higher difficulty for men to fully recognize themselves as being victims of this type of violence (Huntley et al., 2019; Machado et al., 2024). Unfortunately, information regarding the duration of perpetrators’ probation at the time of their participation in the present study was unavailable, as well as any other data enabling the detection of which partners (victims or perpetrators) provided the most reliable reports on IPV and why. Either way, it is noteworthy that women recognize having perpetrated IPV in non-neglectable proportions of occurrences and frequencies. This happens, even though they had been identified as a victim in a legal process, pointing to the complexity of IPV dynamics. Furthermore, this is critical information for risk management as research has shown that couples in which both partners resort to violence (regardless of the reasons) are at greater risk of injury and continuing or escalating violence (Straus & Gozjolko, 2016; Whitaker et al., 2007). Relying solely on one partner’s report may lead to dismissal or miss critical information. Future research should focus on these dynamics, including the motives behind perpetration by each partner, as well as potential predictors of interpartner agreement previously mentioned.

Findings from this study highlight the complexity of IPV assessment in legal settings and call for the need to use both partner reports in these settings, as encouraged by Heckert and Gondolf (2000). Indeed, the definition of what would be a satisfactory level of agreement between partners could be challenging (O’Leary & Williams, 2006). Even in community samples, and considering the agreement on other behaviors beyond the scope of IPV (e.g., Capinha et al., 2024; Marshall et al., 2011; O’Leary & Williams, 2006), it is unlikely that either partner would provide a perfectly accurate report of the behaviors occurring between them. At the very least, measurement error must be accounted for (Moffitt et al., 1997). Different authors (e.g., O’Leary & Williams, 2006; Straus et al., 1996; Yoshikawa et al., 2021) have proposed and/or used the upper-bound estimate, which consists of using the highest of the partner’s reports as representing the true value of IPV between the couple. Nonetheless, as highlighted by Capinha et al. (2024), this would not necessarily lead to better or more accurate estimates of IPV and, at least in research settings, more sophisticated methodologies for statistical analysis—that use both partners’ reports and account for differences and interdependence in those reports—should be preferred. In legal settings, however, an important advantage of the upper-bound method can be identified: when assessing risk, one should minimize the chance of a false negative and/or of underestimating the severity and frequency of violence. This would carry serious consequences for IPV victims and could influence decisions regarding victims’ safety plans or the application of legal sanctions (e.g., protective measures).

Of major relevance would be the need for treatment efficacy studies to rely on more than the assessment of IPV behaviors, as partners do not seem to highly agree in their report about them (particularly, about frequency). Dynamic risk factors (Andrews & Bonta, 2017) that could predispose to engage in these dysfunctional dynamics (both at individual and dyadic levels) should be addressed, implying the need for a clear theoretical framework underlying IPV interventions (McGuire, 2013). The use of police records, a strategy usually followed to assess recidivism after IPV interventions (regarded as a measure of treatment efficacy), may also not be an accurate and valid measure to assess IPV behaviors. This strategy was used by Heckert and Gondolf (2000) and led the authors to conclude that both men and women were underreporting IPV at the beginning of the intervention. However, police reports, as well as the testimony of other people about an IPV incident, are equally subject to the influence of societal attitudes (particularly to a gendered view of IPV as being perpetrated by men, while women are mostly victims; Bates, Kaye, et al., 2019; Bates, Klement, et al., 2019). These attitudes have been found to prevent a valid report and to impact how the police, services, and people in general, respond to an IPV event (Bates, Klement, et al., 2019). Indeed, perpetration by men has been shown as more likely to be identified and reported than perpetration by women (Bates, Kaye, et al., 2019).

Therefore, combining different sources of information and considering the differences in their reports may be the only reliable strategy to ensure the full validity of findings regarding IPV. It is important to remember that interpartner agreement does not assess the credibility of testimony, nor does it serve as its replacement. IPV assessment in a forensic setting should be complemented with various strategies (e.g., forensic interviews) and other instruments focused on different dimensions not covered by the CTS-2 (e.g., controlling behaviors, stalking, financial abuse).

Although some limitations of this study have been already discussed (e.g., not controlling for how long the perpetrator was subjected to a probation measure at the time of data collection), others need to be acknowledged when interpreting the findings herein reported. First, this study only includes intact couples (i.e., couples who remain in an intimate relationship after a judicial intervention), in which the perpetrator was not imprisoned. This could have led to the exclusion of more severe cases of IPV, as more extreme cases could have been associated with a higher likelihood of divorce or dissolution of the intimate relationship, as well as effective prison sentences. Even though it is generally assumed that forensic samples tend to present more severe and coercive-controlling patterns than community samples (Cares et al., 2024; Johnson, 1995, 2008), no questions regarding motivation, controlling behaviors, or fear inflicted on the victims were asked. This prevented an empirically based characterization of the IPV patterns that could be present within this sample (Eckstein, 2017). In addition, the low reliabilities of the injury subscales, although it does not seem to have harmed the agreement levels on those dimensions, should impose extra caution when interpreting those findings and prevent any attempt at generalization.

Despite its limitations, findings from the present study offer empirical support for the use of the CTS-2 as a relevant and valid instrument to assess IPV among different-sex couples in legal settings, as the agreement indexes were superior to those found resorting to other measures. Nonetheless, considering that the levels of agreement had some discrepancies across different types of IPV and depending on the selected indexes, these findings do not provide certainty that the proxy method is entirely reliable in these settings. Being the first work assessing agreement between partners in forensic samples using the CTS-2, findings from this study should be replicated, preferably in larger samples. Bearing in mind the aforementioned risks and also the endorsement of several authors for using reports from both partners (e.g., Cunradi et al., 2009; Moffitt et al., 1997; Yoshikawa et al., 2021), such a strategy is strongly recommended. Future research should also include dyads that are no longer in an intimate relationship or include an analysis of patterns of violence (e.g., using Johnson’s typology; Johnson, 1995, 2008) to investigate if interpartner agreement varies according to it. Different-sex couples in which the woman is the one having a probation measure due to an IPV-related crime toward her current partner, as well as same-sex couples, should also be included in future studies. With very few exceptions (e.g., Walsh & Stephenson, 2023), these populations have been disregarded in the assessment of interpartner agreement and the current study findings may not be representative of their experiences. Likewise, the association between intimate partner agreement and the ethnic or cultural background of the participants should also be explored in future research.

This work represents an important initial contribution to enhancing the understanding of interpartner agreement on IPV reports using the CTS-2 in legal settings, helping practitioners and researchers to support their decision-making processes regarding IPV assessment.

## References

[bibr1-08862605241249740] Andrews D. Bonta J. (2017). The psychology of criminal conduct. Routledge.

[bibr2-08862605241249740] Armstrong T. Wernke J. Medina K. Schafer J. (2002). Do partners agree about the occurrence of intimate partner violence? A review of the current literature. Trauma, Violence, and Abuse, 3(3), 181–193. 10.1177/15248380020033002

[bibr3-08862605241249740] Bates E. Kaye L. Pennington C. Hamlin I. (2019). What about the male victims? Exploring the impact of gender stereotyping on implicit attitudes and behavioural intentions associated with intimate partner violence. Sex Roles, 81(1–2), 1–15. 10.1007/S11199-018-0949-X

[bibr4-08862605241249740] Bates E. Klement K. Kaye L. Pennington C. (2019). The impact of gendered stereotypes on perceptions of violence: A commentary. Sex Roles, 81(1–2), 34–43. 10.1007/s11199-019-01029-9

[bibr5-08862605241249740] Bell K. Naugle A. (2007). Effects of social desirability on students’ self-reporting of partner abuse perpetration and victimization. Violence and Victims, 22(2), 243–256. 10.1891/088667007780477348 17479559

[bibr6-08862605241249740] Botsch R. (2011). Significance and measures of association. In Botsch B. (Ed.), Scopes and methods of political science (pp. 1–7). University of South Carolina.

[bibr7-08862605241249740] Btoush R. Campbell J. (2009). Ethical conduct in intimate partner violence research: Challenges and strategies. Nursing Outlook, 57(4), 210–216. 10.1016/j.outlook.2008.10.005 19631063

[bibr8-08862605241249740] Caetano R. Field C. Ramisetty-Mikler S. Lipsky S. (2009). Agreement on reporting of physical, psychological, and sexual violence among white, black, and Hispanic couples in the U.S. Journal of Interpersonal Violence, 24(8), 1318–1337. 10.1177/0886260508322181 18768744 PMC2705474

[bibr9-08862605241249740] Capinha M. Rijo D. Matos M. Pereira M. (2024). Interpartner agreement on intimate partner violence reports: Evidence from a community sample of different-sex couples. Assessment, 31(5), 980–993. 10.1177/10731911231196483 37732644 PMC11135001

[bibr10-08862605241249740] Capinha M. Rijo D. Pereira M. Matos M. (2022). The prevalence, directionality, and dyadic perpetration types of intimate partner violence in a community sample in Portugal: A gender-inclusive inquiry. European Journal on Criminal Policy and Research. Advance online publication. 10.1007/S10610-022-09514-W PMC9170125 35692937

[bibr11-08862605241249740] Cares A. Khallouq B. Mukazhanova-Powell K . (2024). The reach of Johnson’s typology of intimate partner violence: A scoping review of empirical research, 1996–2020. Journal of Family Violence, 39, 9–21. 10.1007/s10896-023-00602-x

[bibr12-08862605241249740] Cunradi C. Bersamin M. Ames G. (2009). Agreement on intimate partner violence among a sample of blue-collar couples. Journal of Interpersonal Violence, 24(4), 551–568. 10.1177/0886260508317189 18430971 PMC3157480

[bibr13-08862605241249740] Dettori J. Norvell D. (2020). Kappa and beyond: Is there agreement? Global Spine Journal, 10(4), 499–501. 10.1177/2192568220911648 32435572 PMC7222679

[bibr14-08862605241249740] Eckstein J. (2017). Intimate terrorism and situational couple violence: Classification variability across five methods to distinguish Johnson’s violent relationship types. Violence and Victims, 32(6), 1–22. http://dx.doi.org/10.1891/0886-6708.VV-D-16-00022 10.1891/0886-6708.VV-D-16-00022 29017637

[bibr15-08862605241249740] Ellsberg M. Heise L. (2002). Bearing witness: Ethics in domestic violence research. Lancet, 359(9317), 1599–1604. 10.1016/S0140-6736(02)08521-5 12047984

[bibr16-08862605241249740] Esquivel-Santoveña E. Lambert T. Hamel J. (2013). Partner abuse worldwide. Partner Abuse, 4(1), 6–75. 10.1891/1946-6560.4.1.6

[bibr17-08862605241249740] European Institute for Gender Equality. (2014). Estimating the costs of gender-based violence in the European Union: Report. https://eige.europa.eu/publications-resources/publications/estimating-costs-gender-based-violence-european-union-report

[bibr18-08862605241249740] European Union Agency for Fundamental Rights. (2014). Violence against women: An EU-wide survey—Main results. https://fra.europa.eu/en/publication/2014/violence-against-women-eu-wide-survey-main-results-report

[bibr19-08862605241249740] Furr R. (2010). The double-entry intraclass correlation as an index of profile similarity: Meaning, limitations, and alternatives. Journal of Personality Assessment, 92(1), 1–15. 10.1080/00223890903379134 20013451

[bibr20-08862605241249740] Giraudeau B. (1996). Negative values of the intraclass correlation coefficient are not theoretically possible. Journal of Clinical Epidemiology, 49(10), 1205–1206. 8827004 10.1016/0895-4356(96)00053-4

[bibr21-08862605241249740] Graña J. Cuenca M. Redondo N. (2017). Relationship satisfaction and interpartner agreement about acts of physical and psychological aggression: A multilevel analysis. BMC Psychiatry, 17(1), 1–9. 10.1186/S12888-017-1452-6 28810854 PMC5558665

[bibr22-08862605241249740] Gwet K. (2014). Handbook of inter-rater reliability: The definitive guide to measuring the extent of agreement among multiple raters (4th ed.). Advanced Analytics, LLC.

[bibr23-08862605241249740] Hair J. Celsi M. Money A. Samouel P. Page M. (2015). The essentials of business research methods (3rd ed.). Taylor & Francis Inc.

[bibr24-08862605241249740] Heckert D. Gondolf E. (2000). Assessing assault self-reports by batterer program participants and their partners. Journal of Family Violence, 15(2), 181–197. 10.1023/A:1007594928605

[bibr25-08862605241249740] Huntley A. Potter L. Williamson E. Malpass A. Szilassy E. Feder G. (2019). Help-seeking by male victims of domestic violence and abuse: A systematic review and qualitative evidence synthesis. BMJ Open, 9(6), Article e021960. 10.1136/bmjopen-2018-021960 PMC6585830 31186243

[bibr26-08862605241249740] Johnson M. P. (1995). Patriarchal terrorism and common couple violence: Two forms of violence against women. Journal of Marriage and the Family, 57(2), 283–294. 10.2307/353683

[bibr27-08862605241249740] Johnson M. P. (2008). A typology of domestic violence: Intimate terrorism, violent resistance, and situational couple violence. University Press of New England.

[bibr28-08862605241249740] Kimmel M. (2002). “Gender symmetry” in domestic violence. Violence Against Women, 8(11), 1332–1363. 10.1177/107780102237407

[bibr29-08862605241249740] Konstantinidis M. Le L. Gao X. (2022). An empirical comparative assessment of inter-rater agreement of binary outcomes and multiple raters. Symmetry, 14(2), 262. 10.3390/SYM14020262

[bibr30-08862605241249740] Koo T. Li M. (2016). A guideline of selecting and reporting intraclass correlation coefficients for reliability research. Journal of Chiropractic Medicine, 15(2), 155–163. 10.1016/J.JCM.2016.02.012 27330520 PMC4913118

[bibr31-08862605241249740] Krebs C. Breiding M. Browne A. Warner T. (2011). The association between different types of intimate partner violence experienced by women. Journal of Family Violence, 26(6), 487–500. 10.1007/S10896-011-9383-3

[bibr32-08862605241249740] LaMotte A. Taft C. Reardon A. Miller M. (2014). Agreement between veteran and partner reports of intimate partner aggression. Psychological Assessment, 26(4), 1369–1374. 10.1037/PAS0000018 25265413 PMC4274234

[bibr33-08862605241249740] LaMotte A. Taft C. Weatherill R. Scott J. Eckhardt C. (2014). Examining intimate partner aggression assessment among returning veterans and their partners. Psychological Assessment, 26(1), 8–15. 10.1037/a0034579 24079959

[bibr34-08862605241249740] Machado A. Mesquita C. Matos M. (2024). Brief report on a systematic review of the experiences of male victims of intimate partner violence as help-seekers. Journal of Aggression, Maltreatment & Trauma, 33(1), 109–122. 10.1080/10926771.2022.2112336

[bibr35-08862605241249740] Marshall A. Holtzworth-Munroe A. (2002). Varying forms of husband sexual aggression: Predictors and subgroup differences. Journal of Family Psychology, 16(3), 286–296. 10.1037/0893-3200.16.3.286 12238411

[bibr36-08862605241249740] Marshall A. Panuzio J. Makin-Byrd K. Taft C. Holtzworth-Munroe A. (2011). A multilevel examination of interpartner intimate partner violence and psychological aggression reporting concordance. Behavior Therapy, 42(3), 364–377. 10.1016/j.beth.2010.09.003 21658520 PMC3490433

[bibr37-08862605241249740] McGuire J. (2013). “What works” to reduce re-offending: 18 years on. In Craig L. Dixon L. Gannon T. (Eds.), What works in offender rehabilitation: An evidence-based approach to assessment and treatment (pp. 20–49). Wiley-Blackwell.

[bibr38-08862605241249740] Moffitt T. Caspi A. Krueger R. Magdol L. Margolin G. Silva P. Sydney R. (1997). Do partners agree about abuse in their relationship? A psychometric evaluation of interpartner agreement. Psychological Assessment, 9(1), 47–56. 10.1037/1040-3590.9.1.47

[bibr39-08862605241249740] Nunnally J. Bernstein I. (1994). The assessment of reliability. Psychometric Theory, 3, 248–292.

[bibr40-08862605241249740] O’Leary K. Williams M. (2006). Agreement about acts of aggression in marriage. Journal of Family Psychology, 20(4), 656–662. 10.1037/0893-3200.20.4.656 17176201

[bibr41-08862605241249740] Paiva C. Figueiredo B. (2006). Portuguese version of “Revised Conflict Tactics Scales”: A validation study. Psicologia: Teoria e Prática, 8(2), 14–39.

[bibr42-08862605241249740] Panuzio J. O’Farrell T. Marshall A. Murphy C. Murphy M. Taft C. (2006). Intimate partner aggression reporting concordance and correlates of agreement among men with alcohol use disorders and their female partners. Assessment, 13(3), 266–279. 10.1177/1073191106287792 16880279

[bibr43-08862605241249740] Romano E. Weegar K. Gallitto E. Zak S. Saini M. (2021). Meta-analysis on interventions for children exposed to intimate partner violence. Trauma, Violence, and Abuse, 22(4), 728–738. 10.1177/1524838019881737 31623532

[bibr44-08862605241249740] Simpson L. Christensen A. (2005). Spousal agreement regarding relationship aggression on the Conflict Tactics Scale-2. Psychological Assessment, 17(4), 423–432. 10.1037/1040-3590.17.4.423 16393009

[bibr45-08862605241249740] Sistema de Segurança Interna. (2021). Relatório Anual de Segurança Interna (RASI)—2020 (Annual homeland security report). Retrieved April 5, 2021, from https://www.portugal.gov.pt/pt/gc22/comunicacao/documento?i=relatorio-anual-de-seguranca-interna-2021

[bibr46-08862605241249740] Stöckl H. Devries K. Rotstein A. Abrahams N. Campbell J. Watts C. Moreno C. G. (2013). The global prevalence of intimate partner homicide: A systematic review. The Lancet, 382(9895), 859–865. 10.1016/S0140-6736(13)61030-2 23791474

[bibr47-08862605241249740] Stover C. Shafai A. Kwinjo S. Farren A. Mazany E. McFaul C. (2022). Agreement between mother and father: Reports of IPV in a sample of child protection referred coparents. Journal of Interpersonal Violence, 38(3–4), 3489–3512. 10.1177/08862605221107055 35673943

[bibr48-08862605241249740] Straus M. (1979). Measuring intrafamily conflict and violence: The Conflict Tactics Scales. Journal of Marriage and the Family, 41(1), 75–88. 10.2307/351733

[bibr49-08862605241249740] Straus M. (2004). Cross-cultural reliability and validity of the Revised Conflict Tactics Scales: A study of university student dating couples in 17 nations. Cross-Cultural Research, 38(4), 407–432. 10.1177/1069397104269543

[bibr50-08862605241249740] Straus M. Gozjolko K. (2016). Concordance between partners in “intimate terrorism”: A comparison of two typologies. Aggression and Violent Behavior, 29, 55–60. 10.1016/j.avb.2016.06.003

[bibr51-08862605241249740] Straus M. Hamby S. Boney-McCoy S. Sugarman D. (1996). The Revised Conflict Tactics Scale. Journal of Family Issues, 17, 283–316. 10.1037/t02126-000

[bibr52-08862605241249740] Straus M. Mickey E. (2012). Reliability, validity, and prevalence of partner violence measured by the Conflict Tactics Scales in male-dominant nations. Aggression and Violent Behavior, 17(5), 463–474. 10.1016/j.avb.2012.06.004

[bibr53-08862605241249740] Vega E. O’Leary K. (2007). Test-retest reliability of the Revised Conflict Tactics Scales (CTS2). Journal of Family Violence, 22(8), 703–708. 10.1007/S10896-007-9118-7

[bibr54-08862605241249740] Walsh A. Stephenson R. (2023). Reporting of intimate partner violence among male couples: Cross-sectional and serial dyadic concordance. Journal of Family Violence, 38(7), 1325–1339. 10.1007/s10896-022-00439-w PMC7413602 32773962

[bibr55-08862605241249740] Whitaker D. Haileyesus T. Swahn M. Saltzman L. (2007). Differences in frequency of violence and reported injury between relationships with reciprocal and nonreciprocal intimate partner violence. American Journal of Public Health, 97(5), 941–947. 10.2105/AJPH.2005.079020 17395835 PMC1854883

[bibr56-08862605241249740] Yoshikawa K. Shakya T. Poudel K. Jimba M. (2021). Agreement on reporting intimate partner violence among Nepalese couples: A cross-sectional study. Journal of Interpersonal Violence, 36(9–10), 4039–4057. 10.1177/0886260518788371 30019604

